# Biodegradation of kerosene: Study of growth optimization and metabolic fate of *P. janthinellum* SDX7

**DOI:** 10.1590/S1517-838246220140112

**Published:** 2015-06-01

**Authors:** Shamiyan R. Khan, J.I. Kumar Nirmal, Rita N. Kumar, Jignasha G. Patel

**Affiliations:** 1 Department of Environmental Science and Technology, Institute of Science and Technology for Advanced Studies and Research, Gujarat, India, Department of Environmental Science and Technology, Institute of Science and Technology for Advanced Studies and Research, Gujarat, India.; 2 Department of Biological and Environmental Sciences, Natubhai V Patel College, Gujarat, India, Department of Biological and Environmental Sciences, Natubhai V Patel College, Gujarat, India.

**Keywords:** *Penicillum janthinellum* SDX7, optimal growth, metabolites, kerosene degradation

## Abstract

*Penicillum janthinellum* SDX7 was isolated from aged petroleum hydrocarbon-affected soil at the site of Anand, Gujarat, India, and was tested for different pH, temperature, agitation and concentrations for optimal growth of the isolate that was capable of degrading upto 95%, 63% and 58% of 1%, 3% and 5% kerosene, respectively, after a period of 16 days, at optimal growth conditions of pH 6.0, 30 °C and 180 rpm agitation. The GC/MS chromatograms revealed that the*n*-alkane fractions are easily degraded; however, the rate might be lower for branched alkanes, *n*-alkylaromatics, cyclic alkanes and polynuclear aromatics. The test doses caused a concentration-dependent depletion of carbohydrates of *P. janthinellum* SDX7 by 3% to 80%, proteins by 4% to 81% and amino acids by 8% to 95% upto 16 days of treatment. The optimal concentration of 3% kerosene resulted in the least reduction of the metabolites of *P. janthinellum* such as carbohydrates, proteins and amino acids with optimal growth compared to 5% and 1% (v/v) kerosene doses on the 12^th^ and 16^th^ day of exposure. Phenols were found to be mounted by 43% to 66% at lower and higher concentrations during the experimental period. Fungal isolate *P. janthinellum* SDX7 was also tested for growth on various xenobiotic compounds.

## Introduction

Kerosene currently has several uses such as aircraft gas turbine and jet fuel for both commercial airlines and the military activities, as heating oil, and as a spray oil to combat insects on agricultural plants. Because of its availability compared to gasoline during wartime, commercial illuminating kerosene was the fuel chosen for the early jet engines. Consequently, the development of commercial jet aircraft following World War II focused primarily on the use of kerosene-type fuels (Irwin *et al.*, 1997). According to the US Coast Guard Emergency Response Notification System, kerosene is one of the most commonly spilled petroleum products containing paraffins (alkanes), cycloparaffins (cycloalkanes), aromatics, and olefins with carbon numbers betweenC9–C20 (Irwin *et al.*, 1997). Large amounts of spills and leaks of petroleum products such as gasoline, diesel, kerosene, and similar compounds have been refined and handled on land every year. Despite careful handling, there is a possibility of introduction into the soil environment. The penetration of hydrocarbons from the top of the soil into the subsoil leads to a direct risk of ground water contamination (Schinner and Margesin, 1997; Watkinson and Morgan, 1989). Although a significant proportion of the compounds in petroleum products are relatively innocuous, a number of lower molecular weight compounds are toxic or mutagenic and require remedial action to restrict environmental damage (Greer *et al.*, 2003)

Microorganisms are powerful alternatives to conventional methods in resolving environmental problems (Bento *et al.*, 2003). Bacteria, yeast and filamentous fungi have been reported as transforming agents due to their ability to degrade a wide range of pollutants because of their ubiquitous nature. Various bacterial genera that have been reported to contain hydrocarbon degrading species include *Pseudomonas, Vibrio, Arthrobacter* and *Bacillus* (Zajic, 1972; Snape *et al.*, 2001). Fungal degradation of defined single hydrocarbons as well as petroleum products has been performed by *Penicillium, Candida, Fusarium, Aspergillus, Articulosporium* (Sugiura *et al.*, 1997; Mukherji *et al.*, 2004). Kumar *et al.* (2013) performed biodegradation of high molecular weight PAHs such asphenanthrene and fluoranthene using cyanobacterial species - *Aulosira fertilissima* ghose. These organisms have been isolated in large numbers from many oil-polluted waters and soils but are found in lesser numbers in uncontaminated environments (Okoh, 2003). Biodegradation is considered to be a major mechanism for the removal of spilled petroleum hydrocarbons from the environment (Braddock and Lindstrom, 2002). Therefore, it is necessary to perform laboratory feasibility tests to determine the effectiveness of biodegradation of kerosene due to the significant effect of the inherent capabilities of the microorganisms, by their ability to overcome the bioavailability limitations in multiphase environmental scenarios and by environmental factors such as pH, temperature, nutrients and electron acceptor availability (Mukherji *et al.*, 2004; Trindade *et al.*, 2005). Less is known about the biodegradability of petroleum commercial products such as kerosene (Gouda *et al.*, 2007). Shamiyan *et al.* (2013a, b) investigated the interactions of soil nutrients with Total Petroleum Hydrocarbons (TPH), with isolation and characterization of different petroleum-degrading fungal isolates. Moreover, no attempt has been made in the proposed work to establish the differential effects of various concentrations of kerosene on biochemical constituents of the fungal isolate *Penicillium janthinellum* SDX7 in liquid media during the biodegradation process. Hence, in the present investigation, an attempt has been made to optimize the fungal isolate at different conditions and its biochemical response during the petroleum hydrocarbon biodegradation of kerosene.

## Materials and Methods

### Fungal Isolate, Media and Culture Conditions

A fungal strain *P. janthinellum* SDX7 was isolated and screened from aged petroleum hydrocarbon-infected soil at the site of Anand, Gujarat, India, supporting the data of the TPH in the contaminated soil compared to the uncontaminated soil (garden soil). The isolate was identified as *P. janthinellum* SDX7 based on morphological and molecular (18S rRNA) methods, and the sequences were submitted to the National Center for Biotechnology Information (NCBI) gene bank under accession no. KC545842-KC545843 (Shamiyan *et al.*, 2013b). Two types of media were used for cultivation, Potato Dextrose Agar (PDA) media and for the optimization and degradation studies, Mineral Salt Medium (MSM) with the addition of various concentrations of kerosene as the sole carbon source. The liquid medium was sterilized at 121°C for 15 min before addition of the kerosene (Zajic and Supplisson, 1972).

### Preparation of standardized inocula

Axenic spore suspensions of *P. janthinellum* SDX7 of approximately 105 spores/mLwere grown by adding 15 mL of sterile distilled water to mature fungal colonies on PDA plates (4–5 days) to dislodge the spores from the mycelium. These suspensions were then used to inoculate 100 mL MSM containing 3% (w/v) glucose in 500 mL Erlenmeyer flasks (Kendrick and Ratledge, 1996). The cultures were incubated at 30 °C in an incubator shaker operating at 180 rpm for 48 h. The resultant active growing cultures were aseptically washed three times with 300 mL of sterile distilled water to remove remaining glucose fractions. This resulting culture was then used as the standard inoculum for further experiments.

### Determination of optimal growth

The optimization study addresses different pH values, temperature, speed of agitation and kerosene concentration on *P. janthinellum* SDX7. A total of 10% (v/v) of standard inoculum was inoculated in each experiment and performed in triplicate. Biomass production (g/L) was used as an indicator for growth after 7 days of incubation. MSM medium with kerosene (without inoculation) was used as a control (Hamzah *et al.*, 2012).

### Growth parameters

#### pH

The influence of the pH of the initial medium on the fungal growth of *P. janthinellum* SDX7 was determined at pH 4.0, 5.0, 6.0, 7.0, 8.0 and 9.0. Ten percent (v/v) standard inoculum was inoculated in a 500 mL Erlenmeyer flask containing 100 mL of MSM with the addition of 3% (v/v) kerosene and incubated at 30 °C in an orbital shaker at 180 rpm for 7 days. The pH that promoted the highest biomass production (in terms of dry weight) was used for subsequent steps of the investigation.

#### Temperature

The effect of temperature on *P. janthinellum* SDX7 growth was studied at 20, 30 and 40 °C in the MSM medium with 3% (v/v) kerosene at the determined optimum pH and incubated in an orbital shaker at 180 rpm for 7 days. The temperature that promoted the highest biomass production was used for the subsequent steps of the investigation.

#### Speed of agitation

The effect of different agitation at 130, 180 and 230 rpm during incubation on growth of *P. janthinellum* SDX7 was performed in the MSM medium with 3% (v/v) kerosene at optimum pH using an orbital shaker. Incubation was conducted at the determined optimum pH and temperature. The agitation speed that promoted the highest biomass production was used for the subsequent steps of the investigation.

#### Concentration of kerosene

The isolate of *P. janthinellum* SDX7 was grown in MSM prepared in accordance with the optimum nutrient parameters but supplemented with 1%, 3%, 5%, 10% and 20% (v/v) concentrations of kerosene and incubated at optimum pH, temperature and agitation speed for 7 days.

#### Dry weight measurement

The biomass of *P. janthinellum* SDX7 recovered by filtration using Whatman filter paper (No. 4) was washed with 100 mL chloroform to remove residual kerosene, then dried in the oven at 60 °C overnight, and cooled in a desiccators for 10–20 min prior to weighing.

#### Determination of biodegradation activity

The determination of the biodegradation activity of *Penicillum janthinellum* SDX7 was performed in 100 mL of the MSM medium treated by lower 1%, optimal 3% and higher 5% concentrations of kerosene in a 500 mL Erlenmeyer flask and incubated at 30 °C and agitated at 180 rpm for 16 days. The residual petroleum hydrocarbon was recovered by chloroform extraction at a ratio of 1:1 MSM medium: chloroform (Chaillan *et al.*, 2004). MSM without fungal inoculation was used as the control. Analysis of the fungal biodegradation activity was made using a computerized capillary gas chromatograph with flame ionization detector (GC/FID, Perkin Elmer-Auto System, SICART, V.V. Nagar) equipped with HP 3390A Integrator, split injector (split ratio 20/1) and flame ionization detector set at 300 °C. The carrier gas was nitrogen at a flow rate of 1.5 mL min^−1^. The column was polydimethylsiloxane (length 30 m, internal diameter 0.32 mm, film thickness 0.25 μm). The temperature was programmed to increase from 60 to 320 °C at 4 °C min^−1^. The total petroleum hydrocarbon (TPH) degradation by *P. janthinellum* SDX7 isolate was calculated according to the following equation:

(1) $$\%\text{B}=\frac{100\;(\text{TPHC}-\text{TPHI})}{\text{TPHC}}$$

where B is biodegradation, TPHC is the total petroleum hydrocarbon in the abiotic control (without fungal inoculation) and TPHI is the total petroleum hydrocarbon with inoculation (in this case with *P. janthinellum* SDX7).

#### GC/MS analysis

GC/MS spectra were acquired in the electron ionization mode (70 eV, nominal) scanning from *m/z* 30 to 650 s^−1^ and detected using an auto system XL GC apparatus (Perkin Elmer, SICART, V.V. Nagar). The column temperature was initially 80 °C, held for 5 min, then ramped from 80 °C- 290 °C at 10 °C min^−1^. Helium (1.0 mL min^−1^) was used as the carrier gas. Both line and injector temperatures were set at 250 °C. Each methanolic extract (1 μL) was injected in the split mode (1:40). MS conditions were EI + through a Perkin Elmer Turbo mass spectrometer as follows: ionization energy −70 e V, nominal; scan rate, 1.6 scans/s; inter-scan delay, 0.01 s; source temperature, 250 °C; mass range, 30 to 650 Daltons; solvent delay, 3.00 min. The gas chromatogram as reproduced by the mass spectrometer identified the mass spectrum scanned at each GC peak maximum. Data were thus obtained by comparing the mass spectra to those in the Wiley NIST/EPA/NIH Mass Spectral Library 2005.

#### Determination of metabolites

The variation in the metabolites was determined on the 4^th^, 8^th^, 12^th^ and 16^th^ days by cultivating the *P. janthinellum* SDX7 culture under optimal conditions such as pH 6, temperature of 30 °C, speed of agitation at 180 rpm and addition of optimal 3% (v/v) with higher 5% and lower 1% (v/v) dose of kerosene. Total carbohydrate release was determined spectrophotometrically by the anthrone method using glucose as the standard (Hedge and Hofreitte, 1991). The protein content of the crude cell-free extract was estimated (Lowry *et al.*, 1951) using bovine serum albumin as the standard. An improved colorimetric determination of amino acids by the use of ninhydrin was performed (Lee and Takahashi, 1966). Phenol stress metabolites were estimated using Folin-Ciocalteu reagent (Malick and Singh, 1980). Each experiment was conducted in replicates of three and their ± SE values were calculated. Multivariate analysis was performed using KY plot (2.0 beta) to estimate the correlation between metabolites.

#### Growth of P. janthinellum SDX7 on other xenobiotic compounds

Fungal isolate *P. janthinellum* SDX7 was also tested for its growth on various xenobiotic compounds, namely, diesel fuel, gasoline fuel, toluene, benzene, xylene, naphthalene, pyrene and phenanthrene at a concentration of 0.1% [(w/v)/ (v/v)] as a sole carbon source in MSM and incubated under optimized conditions (at 30 °C temperature and shaking conditions of 180 rpm). The samples were withdrawn after 7 days, and the biomass was estimated in the form of the dry weight (Gojgic *et al.*, 2012).

## Results and Discussion

### Optimization of growth parameters

**Figure 1:**
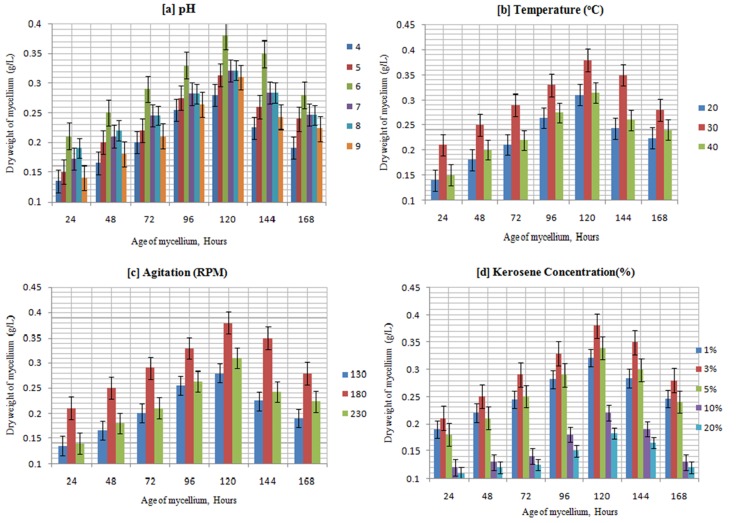
Optimal biomass production of *P. janthinellum SDX7* after 7 days of incubation.

*P. janthinellum* SDX7 grew better under acidic conditions, showing optimal growth (0.38 g/L) at pH 6. Previous studies also reported that several fungal isolates such as *Fusariumsolani*, *F. oxysporum*, *Trichodermaviride* (Verdin *et al.*, 2004) and *Aspergillus niger* (Srivastava and Thakur, 2006) cultured in acidic MSM medium provided good growth. Although its growth was highest under acidic conditions, isolate *P. janthinellum* SDX7 was able to grow sparsely in a relatively wide range of pH values from 4.0 to 9.0, suggesting that this isolate could degrade kerosene under not only acidic but also under neutral and alkaline conditions (Figure 1a). Among the parameters that could affect biomass production, temperature was generally considered to be the most important and limiting factor (Delille, 2004). The common incubation temperature for the growth of fungi such as *A. niger*, *Fusarium sp., Penicillium sp.* and *Graphium sp*. is taken to be 30 °C (Santos and Linardi, 2004). Moreover, in this study, *P. janthinellum* SDX7 cultured at different temperatures produced maximum biomass (0.38 g/L) when incubated at 30 °C compared to 20 °C and 40 °C, temperatures that resulted in the production of a maximum of 0.31 g/L and 0.33 g/L biomass, respectively (Figure 1b). This difference may result from a greater production of enzymes and optimal growth conditions of the isolate for its kerosene degradation (Rohilla *et al.*, 2012). This range of temperatures makes this isolate suitable for use in bioremediation in tropical climates. *P. janthinellum* SDX7 showed an increase of biomass as the rate of agitation increased up to 180 rpm; biomass production was then reduced when the speed of agitation accelerated to 230 rpm (Figure 1c). Agitation influenced the tested fungi to absorb more nutrients by not only increasing the surface area of the microorganism for degradation of the kerosene oil hydrocarbons (Lee *et al.*, 1996) but also by booming the amount of dissolved oxygen in the cultivation medium (Purwanto *et al.*, 2009). Agitation speed has also been proven to be a critical factor influencing mycelial biomass (Hamzah *et al.*, 2012). *P. janthinellum* was found to grow in a wide range of kerosene concentrations. The production of biomass rose to 0.38 g/L in the presence of 3% (v/v) of kerosene on the 5^th^ day. However, the production of biomass declined when the concentration of kerosene escalated beyond 3% (v/v) (Figure 1d). Notably, minimal growth was found when the medium contained 10% and 20% kerosene doses.

### Biodegradation of kerosene

**Figure 2:**
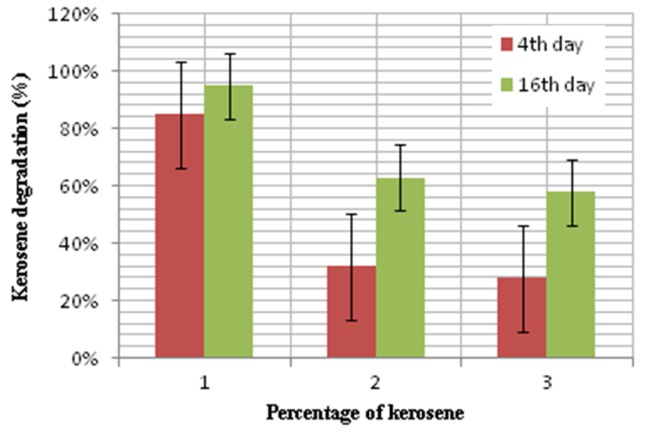
Degradation of different concentrations of kerosene after 4^th^ and 16^th^ days by *P. janthinellum SDX7.*

**Figure 3:**
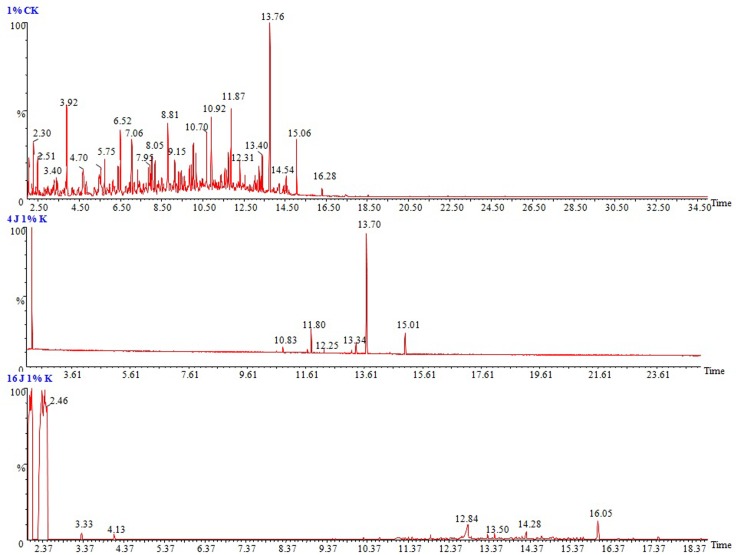
GC/MS chromatograms of 1% (v/v) kerosene in MSM medium. (a) Abiotic control, (b) After 4 days of incubation with *P. janthinellum SDX7* strain, and (c) After 16 days incubation with *P. janthinellum SDX7* strain. The sharp and highest peaks stand for the *n*-alkanes, and the peaks between them comprise the naphthenes and aromatics.

**Figure 4:**
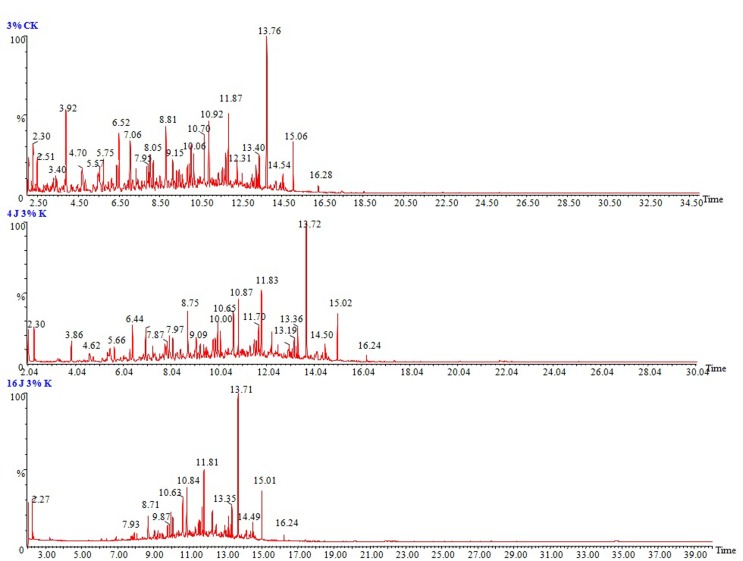
GC/MS chromatograms of 3% (v/v) kerosene in MSM medium. (a) Abiotic control, (b) After 4 days of incubation with *P. janthinellum SDX7* strain, and (c) After 16 days incubation with *P. janthinellum SDX7* strain.

**Figure 5:**
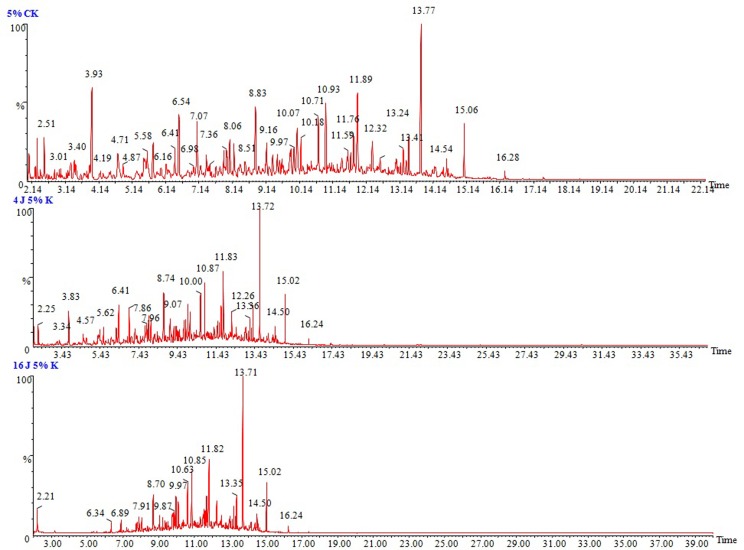
GC/MS chromatograms of 5% (v/v) kerosene in MSM medium. (a) Abiotic control, (b) After 4 days of incubation with *P. janthinellum SDX7* strain, and (c) After 16 days of incubation with *P. janthinellum SDX7* strain.

To determine the ability of *P. janthinellum* SDX7 to degrade kerosene, the total petroleum hydrocarbons were estimated at the end of the 4^th^ and the 16^th^ day of treatment at 1%, 3% and 5% kerosene treatments. Where 1% v/v kerosene showed the highest degradation at 85% and 95%, followed by 3% kerosene treatment at 32% and 63% degradation and a 5% kerosene dose by 28% and 58% reduction on 4^th^ and 16^th^ day, respectively (Figure 2). The biodegradation of kerosene was confirmed by the reduction in the area under the hydrocarbon peaks of the chromatograms when compared to that of the abiotic control (without organism), suggesting that the removal of kerosene hydrocarbon components ranged from 8 to 18 carbon atoms. In the chromatographic images of 1%, 3% and 5% (v/v) kerosene doses in a liquid medium, the sharp and highest peak stands for the *n*-alkanes, and the peaks between them comprise the naphthenes and aromatics. *P. janthinellum* SDX7 showed a reduction in the area under the hydrocarbon peaks corresponding to the carbon atoms (C8 to C18) when compared to the abiotic control in the case of the 1%, 3% and 5% kerosene, where the lowest concentration of 1% displayed the highest reduction (Figure 3) followed by 3% (Figure 4) and 5% (Figure 5). The chromatograms showed that the *n*-alkane fractions are easily degraded by the tested fungal isolate as the days progress. However, the rate might be lower for branched alkanes followed by *n*-alkylaromatics, cyclic alkanes and polynuclear aromatics. The present results are in agreement with the findings obtained by Wang *et al.* (1998), who studied the comparison of crude oil composition changes due to biodegradation and physical weathering using different fungal and bacterial isolates. Moreover, Nocentini *et al.* (2000) studied the biotreatability and feasibility of a bioremediation process by a bacterial species for a kerosene-contaminated soil. The results indicated that the *P. janthinellum* SDX7 isolate in this study is extremely efficient in degrading kerosene hydrocarbons. Our results also agreed very well with the findings of Mancera-López *et al.* (2008), who reported that the *Penicillum* genus was one of the major hydrocarbon-degrading groups.

### Metabolites

**Figure 6:**
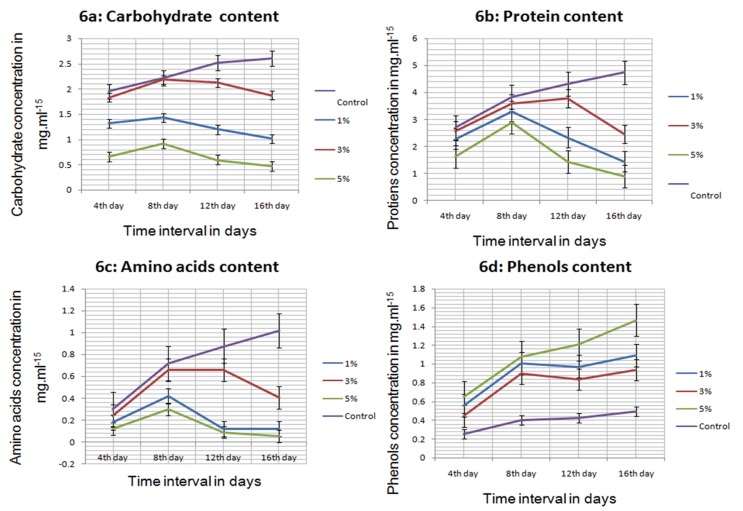
Metabolic content in *P. janthinellum SDX7* treated with kerosene at different daily intervals.

Based on the inhibitory effects and growth arrest, the release of certain metabolic products such as carbohydrates, amino acids and proteins was affected at the earlier stage of kerosene applications, a result that is in agreement with the study of Kumar *et al.* (2013) who studied the impact of PAH exposure on certain cyanobacterial species. The level of total carbohydrates decreased after 12 to 16 days in all the concentrations of kerosene-treated isolate. After the 12^th^ day of exposure, carbohydrate content ranged from 0.6 mgmL^−15^ to 2.24 mgmL^−15^ and was significantly reduced by 50%, 11% and 76%, whereas a higher level of reduction was encountered after the 16^th^ day by 59%, 24% and 80% at 1%, 3% and 5% kerosene exposures to *P. janthinellum* SDX7, respectively (Figure 6a). A similar reduction in the carbohydrate content was recorded by Kumar *et al.* (2008) in nitrogen-fixing cyanobacteria when treated with endosulfan*.* Protein content fluctuated from 1.44 mgmL^−15^ to 3.78 mgmL^−15^ and was inhibited by 46%, 13% and 67% on the 12^th^ day (Figure 6b) and 70%, 48% and 81% after the 16^th^ day, significantly at 1%, 3% and 5%, respectively, of kerosene-treated *P. janthinellum* SDX7 isolate as shown by Babu *et al.* (2001) and Laxmi *et al.* (2007) in response to lindane and organophosphorus on certain cyanobacterial species. At the end of the experiment after the 16^th^ day, the highest reduction of amino acids in *P. janthinellum* SDX7 (by 95%) was observed when treated with 5% kerosene relative to the control, which ranged from 0.05 mgmL^−15^ to 0.4 mgmL^−15^ (Figure 6c). The optimal concentration of 3% kerosene showed less impact on the metabolite reduction. However, the greatest reduction observed at the higher concentration of 5% followed by the lower concentration of 1% kerosene showed results quite well correlated with the findings of Standyk *et al.* (1971), who depicted concentration-dependent inhibition of amino acids and proteins at an earlier stage based on inhibitory effects and growth arrest in fresh water algae in response to pesticide treatments.

A more pronounced effect of kerosene on phenol content was observed on the tested fungal isolate. The release of the stress metabolite phenol was significantly stimulated after 4days of exposure, varying from 0.4 mgmL^−15^ to 0.64 mgmL^−15^ and higher in all treated cultures compared to untreated isolate (Figure 6d). The highest level of phenol was observed in 5% treated isolates, showing 61%, 63%, 65% and 66% increase on the 4^th^, 8^th^, 12^th^ and 16^th^ days, respectively. Elevated levels of phenols might be due to the liberation of the phenolic compounds during gradual degradation of kerosene by hydrolysis or oxidation processes and release under stress conditions because of the catabolic activity of primary metabolites (Mostafa and Helling, 2002). Kumar and Kumar (2002) also suggested that the release of phenols due to applications of different concentrations of fungicide was higher than untreated cultures, possibly due to the accumulation of phenolic compounds from the larger polycyclic aromatic compounds.

A significant positive correlation between carbohydrates, proteins and amino acids (r = 0.88 to 0.94) was encountered, whereas a highly negative correlation was registered with phenols (r= −0.48 to −0.72) after 16 days of exposure to kerosene (Table 1).

**Table 1:** Correlation matrix for *P. janthinellum* SDX7 after 16days of exposure to kerosene.

	Carbohydrates	Proteins	Amino acids	Phenols
Carbohydrates	1			
Proteins	0.88	1		
Amino acids	0.88	0.94	1	
Phenols	−0.72	−0.56	−0.48	1

### Growth of *P. janthinellum* SDX7 on various xenobiotic compounds

The efficiency of the fungal isolate in utilizing other xenobiotic compounds as the sole source of carbon is represented in Table 2. *P. janthinellum* SDX7 displayed a very high growth in the presence of kerosene, diesel and gasoline fuels. However, a moderate growth was encountered in case of naphthalene and poor growth was registered for phenanthrene, toluene, benzene and phenol. The isolate was not able to utilize the high molecular weight PAHs such as pyrene. Our results have been further substantiated by earlier studies of Okerentugba and Ezeronye (2003), revealing the degradation of various xenobiotic compounds by *P. janthinellum* SDX7 based on the ability of utilizing these xenobiotic compounds as the sole source of carbon.

**Table 2:** Growth of kerosene-degrading *P. janthinellum* SDX7 in the presence of different xenobiotic compounds (0.1% [(v/v)/ (w/v)].

Substrate	Growth profile
Kerosene	+++
Diesel Fuel	+++
Gasoline Fuel	+++
Naphthalene	++
Phenanthrene	+
Pyrene	−
Benzene	+
Phenol	+
Toluene	+
Xylene	−

+++ Very high growth, ++ moderate growth, + low growth, − no growth.

The present investigation suggests that the fungal isolate *P. janthinellum* SDX7 employed in this study is extremely efficient in degrading kerosene, displaying a maximum degradation of 95% after 16 days under optimal growth conditions based on its ability to utilize kerosene. The optimal kerosene concentration of 3% showed the least reduction of the metabolites- carbohydrates, proteins and amino acids compared to the 1% and 5% (v/v) kerosene doses on the 12^th^ and 16^th^ days of exposure. Stress metabolite phenol was found to rise at lower and higher concentrations due to the inhibitory effect and growth retardation of the test organism in response to kerosene treatments. The highly efficient degradative ability of *P. janthinellum* SDX7 proved to be suitable for mycoremediation of kerosene-contaminated soil environments.
